# Observational and model evidence together support wide-spread exposure to noncompensable heat under continued global warming

**DOI:** 10.1126/sciadv.adg9297

**Published:** 2023-09-08

**Authors:** Carter M. Powis, David Byrne, Zachary Zobel, Kelly N. Gassert, A. C. Lute, Christopher R. Schwalm

**Affiliations:** ^1^ Environmental Change Institute, University of Oxford, Oxford, UK.; ^2^ Woodwell Climate Research Center, Woods Hole, Falmouth, MA, USA.

## Abstract

As our planet warms, a critical research question is when and where temperatures will exceed the limits of what the human body can tolerate. Past modeling efforts have investigated the 35°C wet-bulb threshold, proposed as a theoretical upper limit to survivability taking into account physiological and behavioral adaptation. Here, we conduct an extreme value theory analysis of weather station observations and climate model projections to investigate the emergence of an empirically supported heat compensability limit. We show that the hottest parts of the world already experience these heat extremes on a limited basis and that under moderate continued warming parts of every continent, except Antarctica, will see a rapid increase in their extent and frequency. To conclude, we discuss the consequences of the emergence of this noncompensable heat and the need for incorporating different critical thermal limits into heat adaptation planning.

## Teaser

Analysis of observations and climate models suggest a rapid increase in noncompensable heat under moderate global warming.

## INTRODUCTION

There is a limit to the thermal conditions the human body can survive without cooling assistance (*1*). Given that we live on a warming planet, it is critical to understand whether these limits could be exceeded as a result of changing environmental conditions and, if so, where and when. Sherwood and Huber (*2*) propose one of the most-cited upper limits to survivable environmental conditions for human beings: six hours of exposure to 35°C wet-bulb (not to be confused with the more common wet-bulb globe temperature), which is defined as the temperature to which a parcel of air can be cooled by evaporation at standard atmospheric pressure. Their research used a simplified general circulation model (GCM) with a slab ocean to suggest that a global average temperature increase of 5° to 7°C would be necessary for temperatures to exceed 35°C wet-bulb on Earth on an annual basis (*2*). Later analysis using ensembles of fully dynamic coupled atmospheric-ocean GCMs (AOGCMs) or Earth system models (ESMs) suggested that these temperatures could occur with less-than-annual return periods across some regions, specifically the Persian Gulf and South Asia, given the global average temperature increases of 3° to 4°C, indicating that lethal heat could first emerge near the end of the century under a pathway of substantial continued emissions or high climate sensitivity (*3*–*8*). The application of statistical methods to observational reanalysis and weather station data has also been used to provide a second line of evidence for the emergence of 35°C wet-bulb heat extremes. Reanalysis-based studies demonstrated geographically limited observations already reaching, and even briefly exceeding, the 35°C wet-bulb threshold in the Persian Gulf, with a 34.6°C observation made in July of 2015 and a 35.4°C observation in 2016 (*9*). Furthermore, HadISD weather station data demonstrate that there are 21 weather stations across the globe that have observed maximum wet-bulb temperatures at or above 35°C in the historical record (*10*). Statistical extrapolation of trends in reanalysis data suggests that 35°C wet-bulb temperatures will first emerge at a grid-cell (reanalysis) level over land, given less than 2.5°C of global warming (*10*).

**Fig. 1.**
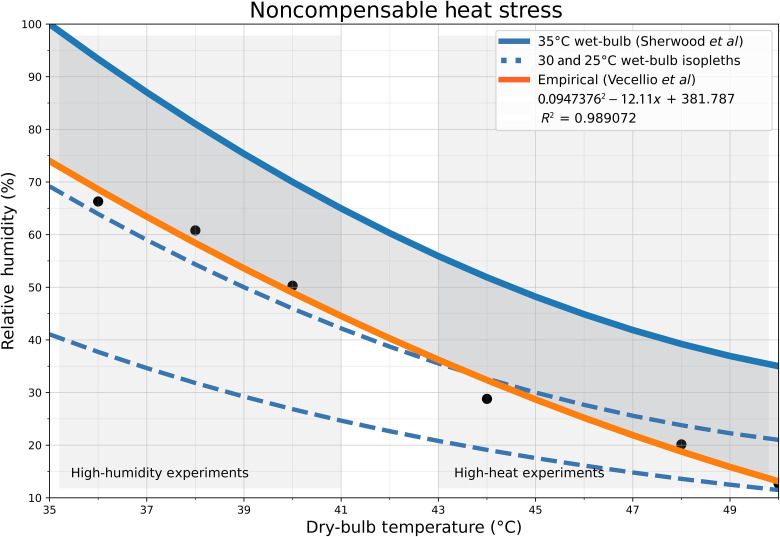
Wet-bulb temperature isopleths and the human noncompensable heat threshold. Wet-bulb temperature isopleths in blue for 35° (bold), 30°, and 25°C (dashed) at standard atmospheric pressure. 35°C wet-bulb is the survivability threshold proposed by Sherwood *et al.* (*2*). A quadratic function fit to empirical evidence for conditions resulting in noncompensable heat stress is displayed in orange. Noncompensable heat stress occurs at points above the orange line and compensable below. Empirical evidence is drawn from two sets of experiments conducted as part of Vecellio *et al.* (*12*): three high-humidity experiments and three high-heat. The average environmental conditions at which heat stress became noncompensable for all participants in each experiment are plotted as points in black. Fit function and *R*^2^ value are in legend.

Sherwood and Huber’s 35°C wet-bulb threshold was proposed as a theoretical upper limit to survivable temperature taking into account maximum physiological and behavioral adaptation. Recent research has demonstrated that the use of a single wet-bulb temperature to determine survivability is an overly simple approach (*11*) and that heat-driven morbidity and mortality outcomes are a function of a number of variables including (i) a much wider and less-extreme range of environmental exposure (*12*), (ii) the physical health and level of heat acclimatization of the exposed population (*13*), and (iii) the availability and efficacy of heat adaptation strategies and tools (*14*–*15*). Here, we make use of laboratory-derived physiological data (Fig. 1) to examine the emergence of an empirically supported heat compensability limit (“noncompensable heat stress”) under various levels of global warming (*12*). Noncompensable heat stress is defined as the set of environmental conditions under which a healthy human being can no longer maintain a stable core temperature without the assistance of external cooling. All else held equal, exposure to 6 hours of noncompensable heat could result in a lethal rise in core temperatures for a healthy human being (see Materials and Methods for more details) (*16*).

It is not possible to link noncompensable heat stress directly to projections of morbidity or mortality due to the same confounding variables discussed with reference to the 35°C wet-bulb survivability threshold. However, it remains a policy-relevant metric because it represents a possible inflection point in the historical relationship between temperature and population morbidity and mortality. Communities are often only prepared for extreme temperature events within the bounds of past experience, and populations only acclimatized to the present climate (*17*). As discussed elsewhere, the likelihood of experiencing abrupt transient temperature extremes well outside of the boundaries of historical experience is increasing everywhere (*17*). Should a noncompensable heat extreme occur in a region where the local population is unprepared and not sufficiently acclimatized, the number of excess deaths that result could markedly exceed the impacts of past local extremes. To understand where and when these noncompensable conditions could occur, we conduct an extreme value analysis of both weather-station observations and bias-corrected and downscaled projections from CMIP6 (Coupled Model Intercomparison Project 6), the latest ensemble of climate models.

## RESULTS

**Fig. 2.**
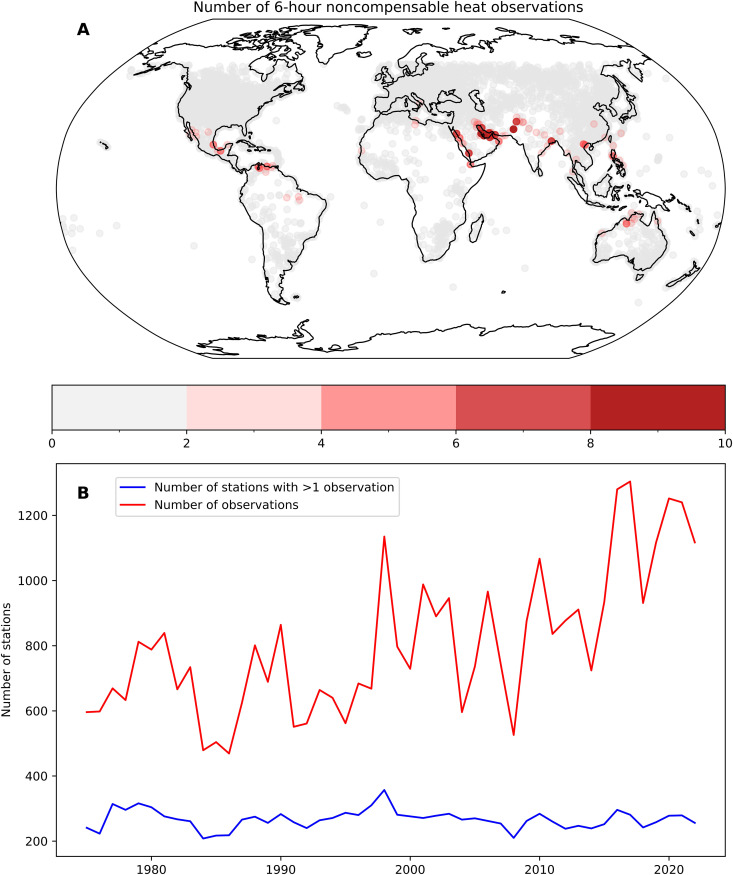
Observations of noncompensable heat in weather station records. (**A**) Total number of 6-hour noncompensable heat observations by weather station between 1970 and 2020. (**B**) Count of total number of weather stations with at least one noncompensable observation between 1970 and 2020 (blue) and count of total number of observations across all stations (red) for the same time period. Data controlled for increase in number of reporting stations by removing all stations with a dataset beginning between 1970 and 2020.

To examine the incidence of noncompensable heat stress in the global historical record, we used HadISD weather station data (v3.3.0.2022f) (*18*) (Fig. 2). There are limited incidences of noncompensable heat stress in weather station records ranging back to at least 1950. Between 1970 and 2020, there were 357 stations across the globe (approximately 4% of total examined) that experienced at least one 6-hour period of noncompensable heat stress. These stations are broadly constrained either to high-heat, high-humidity regions such as Persian Gulf, Red Sea, and the Indo-Gangetic Plain or extreme dry-heat regions such as West Australia and the Sonoran Desert.

The Persian Gulf and Red Sea regularly record the highest humid heat extremes on the planet (*2*, *3*). This region experiences regular clear sky conditions owing to rising air over the monsoon region to the east, causing subsidence and thus suppressing deep convection (*19*). In addition, the region’s surface albedo is low and the major bodies of water shallow, resulting in absorption of solar radiation and increased water vapor and heat retention at the surface, which is moved inland by air currents (*3*). The Indo-Gangetic plain is another region known for its extreme humid heat, driven by the Indian monsoon system transporting warm and humid air masses inland from the Arabian Sea and Bay of Bengal and by substantial levels of local irrigation (*4*). El Niño years are particularly correlated with abnormal heat waves in the region, given delays to the onset of the summer monsoon that allow for a build-up of extreme inland temperatures before the seasonal introduction of moisture and humidity to the region (*20*–*21*). In these regions, noncompensable heat stress is driven by a reduction of the human body’s ability to exhaust heat through the evaporation of sweat, given a reduced vapor pressure differential between the surface of the skin and atmosphere (*2*, *12*).

In contrast, hot-dry regions such as the Sonoran Desert can produce noncompensable heat stress with extreme air temperatures alone. With essentially free evaporation occurring in hot-dry regions, the human body does not increase its sweat rate to compensate for the relatively higher dry heat gains compared to an environment with both extreme temperatures and humidity levels. Hence, noncompensable heat stress tends to occur earlier in hot-dry environments compared to those that are hot-humid (*12*). The Sonoran Desert generates some of the world’s highest air temperatures given its location in a low-elevation basin beneath a persistent subtropical atmospheric ridge and rain shadow. The resulting aridity reduces the region’s ability to exhaust heat in soil via evapotranspiration, and surface heat imbalances are instead compensated for through longwave radiation flux adjustments (*22*–*23*).

To understand how noncompensable heat stress has responded to historical increases in global average temperature, we also examined change in the geographic extent and frequency of observations, controlling for the increase in number of stations over time by removing any station with a dataset beginning between 1970 and 2020 (Fig. 2B). There has been a substantial historical increase in frequency of noncompensable heat as a function of increase in global average temperature, with the annual number of observations increasing on average by about 110 per decade or roughly doubling between 1970 and 2020. Local peaks in both number of stations experiencing noncompensable heat and the frequency of these observations during the El Niño events of 1997, 2010, and 2016 are notable. The number of stations recording at least one 6-hour period of noncompensable heat stress has not yet demonstrated a statistically significant trend over time. Hence, note that only already heat-adapted populations have been exposed to noncompensable heat extremes to date. Given continued increase in air temperatures across the globe, humidity in specific regions, and in the frequency of extreme El Niño events (*24*), we expect both the geographic range and frequency of noncompensable heat to increase going forward, exposing additional populations to increased levels of heat risk and further necessitating comprehensive heat adaptation strategies.

### Statistical extrapolation of weather station observation trends

**Fig. 3.**
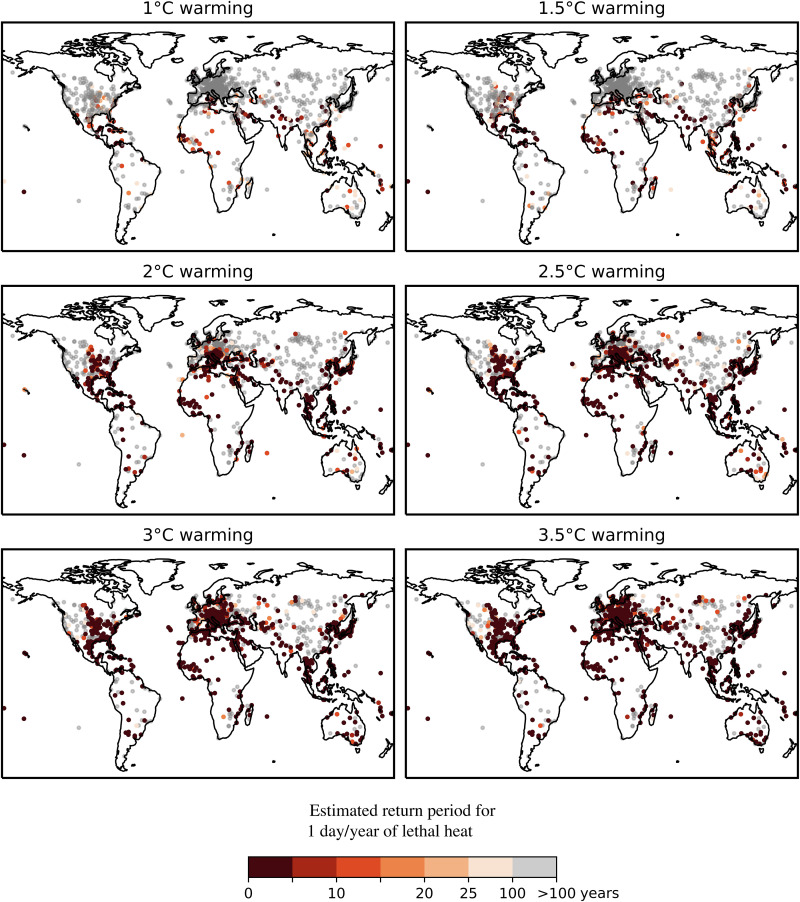
Estimated return periods for at least 6 hours of continuous noncompensable heat stress in a given year. Panels contain results for climate regimes representing 0.5°C increments between a 1° and 3.5°C increase in global average temperature above preindustrial baseline. Return periods estimated by nonstationary GEV extrapolation of observed weather station data between 1970 and 2020. Density of results alone should not be considered a proxy for magnitude of exposure—the density of results is primarily a function of the locations of high-quality weather station data.

To understand how the incidence and range of noncompensable heat extremes could increase under continued planetary warming, we performed a statistical extrapolation of the temperature and humidity trends contained in HadISD weather station data. We did so by identifying the stations exhibiting annual block maxima that could be appropriately described by a nonstationary generalized extreme value (GEV) distribution, wherein the distribution location is parameterized as a linear function of global average temperature (full details in Materials and Methods). Nonstationary GEV distributions have a long history of application to the projection of future climate extremes from both observational and modeled data (*25*–*28*), including the projection of lethal heat extremes (*10*). Estimated return periods were then calculated from each station’s respective GEV distribution for a year with at least 1 day of 6-hour exposure to noncompensable heat stress under six different warming regimes: global average temperatures at 0.5°C increments between 1° and 3.5°C warmer than the preindustrial average (Fig. 3).

Our results demonstrate that under a 1°C increase in global average temperatures, an occurrence of at least 6 hours of noncompensable heat stress is currently less than a once-in-a-century event for the majority of the land surface of the planet or 82.5% of weather stations examined. The exceptions to this rule are found in the world’s most extreme hot-humid or hot-dry regions: the Persian Gulf, the Indo-Gangetic Plain, parts of the Indonesian Archipelago and the east coast of China, the Northern coast of Australia, and parts of coastal Central America. There is also a small cluster of stations in the American Midwest where fitting a nonstationary GEV distribution produces a return period larger than what might be intuited from the number of historical observations alone. Extreme value distributions can take three generalized forms, one of which exhibits a steep right tail. When modeling humid heat extremes, it is common to find that this distribution is the most appropriate fit, given a convective instability threshold that makes loading the atmosphere with additional water impossible without inducing storm activity (*2*). A corollary of this fact is that it takes only a very small adjustment in the location of the mean of such a distribution to drive large increases in return periods of heat extremes. This is the case here, wherein using the GEV fit adds fidelity to a return period calculation that otherwise would be based purely on observations largely sampled outside the current climate state, given the rapid rise in global average temperatures (*26*).

Given only a 2°C increase in global average temperatures, the percentage of stations globally for which noncompensable heat occurs less than once in a century will decline from 82.5 to 63.9%. The percentage of stations for which these extremes become decadal events will increase from 8.1 to 25.3% (note here that these percentages are calculated relative to the subset of stations used for the GEV analysis, not the total HadISD dataset: see Materials and Methods). Outside of the general increase in frequency and geographic range of noncompensable heat stress as the planet warms, two other noteworthy phenomena are the rapid advances of noncompensable heat exposure north within the East Coast and Midwest regions of the United States and north from the Mediterranean into Europe between 1° and 2°C.

**Fig. 4.**
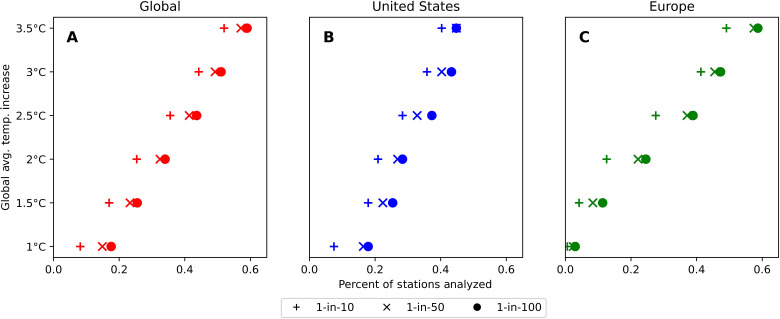
Estimated global and regional return periods for noncompensable heat extremes under different global warming regimes. Panels illustrate the percentage of weather stations exhibiting a given return period for noncompensable heat (**A**) globally, (**B**) in the continental United States, and (**C**) across the European continent, under climate regimes representing 0.5°C increments between a 1° and 3.5°C increase in global average temperature above preindustrial baseline. Return periods estimated by nonstationary GEV extrapolation of a subset of observed weather station data between 1970 and 2020.

Under 1°C warming, only 2.9% of stations in Europe exhibit a return period for a noncompensable heat event more frequent than 1-in-100 years. By 2°C, this has increased to 24.5% and by 3.5°C to 43.4%. Under 2°C, warming noncompensable heat events become a decadal occurrence for 12.6% of European stations and for 49.1% under 3.5°C (Fig. 4). The stations seeing the largest increases in return-period frequency are located in the Balkan states and central Europe, including Germany, Switzerland, and parts of Italy. This marked increase in frequency and extent of noncompensable heat events is in line with current understanding of the dynamics governing European temperature extremes and their evolution under continued global warming. It is well understood, for example, that the hottest European days are warming faster than the mean summer day (*29*) and that extreme heat events in this region are projected to increase disproportionately compared to the global mean temperature in the future (*30*). It has also been demonstrated that the observed magnitude of increases in heat extremes in Europe is not captured appropriately even by the most contemporary ensemble of climate model projections (*31*–*32*). While the exact mechanism for this rapid increase in heat extremes is not yet well understood, several possible phenomena have been proposed: (i) declining early-summer soil moisture (*33*), (ii) a weakened poleward temperature gradient at mid to high latitudes caused by shrinking Arctic sea ice and Eurasian snow cover (*34*), (iii) increased frequency of high pressure over Europe due to a summer slowdown of the Atlantic meridional overturning circulation (*35*), and (iv) the stagnation of ridges and troughs in the mid-latitudes linked to the increased formation of double jets in the troposphere (*32*).

In the United States, under 1°C warming, 17.9% of stations exhibit a noncompensable heat return period more frequent than 1-in-100 years (and 7% return periods more frequent than 1-in-10 years). This percentage increases to 28.3% (20.9%) by 2°C and 44.7% (40.2%) by 3.5°C (Fig. 4). In the hottest locations, noncompensable heat approaches an annual event. Drivers of increases in heat extremes across the United States are highly regionally dependent (*36*) and include changes in the frequency of large-scale anticyclonic circulation patterns due to Arctic amplification (*37*) and changes in land-atmosphere feedbacks including, for example, the strongly negative correlation between atmospheric transient eddies and surface temperature over the Western and Northeastern United States (*36*). Our results suggest that the most marked increase in noncompensable heat extreme frequency will occur across the Great Plains region. Other work has identified a weakening and increase in variability of the Great Plains low-level jet as a key driver of future heat extreme behavior in this region (*38*–*39*). Note that, in the Southern High Plains, present-day rates of groundwater depletion will necessitate a reduction in the scale of irrigation going forward (*40*), which, in turn, could also have substantial impact on the susceptibility of the region to increasing heat extremes and may already be reflected in weather station data (*41*).

Analysis of weather station data offers important benefits when considering how future heat extremes may evolve under a warming climate, the principal of which is their ability to precisely capture local conditions. This offers an advantage over other resources, as trends in temperature extremes are often heavily influenced by hyperlocal environmental factors including geography, vegetation, irrigation, urban heat island/cool island effects, and aerosol concentrations, which are not captured, or not accurately captured, by coarser tools such as observational reanalysis (*10*, *42*–*46*). However, there are also weaknesses of observational data, including the confoundment of long-term climatological trends by short-term trends in the environmental factors, potential biases from observational procedures, instrumentation type, siting, and incomplete spatial coverage, as well as the possibility that local climate will exhibit a nonlinear response to global average change (*10*, *47*). While, here, we have taken efforts to only investigate observational events that show a clear correlation with global average temperature, it is still therefore important to test our station data–based projections with other complimentary lines of evidence. One such possible line of evidence is bias-corrected projections from an ensemble of contemporary climate models, which have been widely and successfully used for analyzing and projecting extreme temperatures (*3*–*5*, *46*, *48*–*49*).

### Climate model projections

**Fig. 5.**
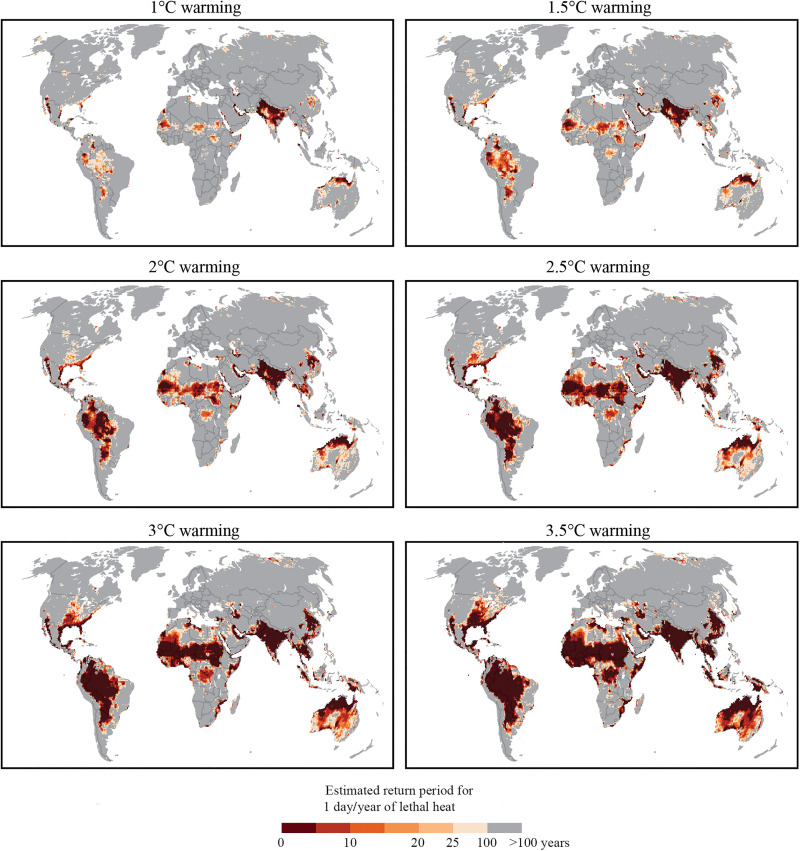
Estimated return periods for at least 6 hours of continuous noncompensable heat stress in a given year. Panels contain results for climate regimes representing 0.5°C increments between a 1° and 3.5°C increase in global average temperature above preindustrial baseline. Return periods estimated using projections from bias-corrected and downscaled CMIP6 ensemble of ESMs.

To investigate whether using fully dynamic process–based models would provide materially different answers to the question of how the range and frequency of noncompensable heat evolve under a warming climate, we examined projections of changing return periods under different levels of global warming directly from 17 bias-corrected and statistically downscaled ESMs/AOGCMs from the CMIP6 ensemble (Fig. 5). In addition to providing an opportunity to compare our weather station–based results to a physical simulation, the other benefit of climate ensemble–derived projections is the availability of 510 years of simulated observations for each warming period, compared to 50 years of direct observation, providing roughly 10× the data from which to extract return period information. As a general rule, the fidelity of return period statistics for extreme heat events increases with the sample size from which they are derived (*50*).

Because climate models do not resolve all local processes that influence weather station–based observations and there are substantial differences in scale between the two datasets (the model data have a downscaled resolution of 0.5° by 0.5° or ~55 km by 55 km at the equator), we expected a priori that return periods should differ between the two projections. Hence, to compare the separate lines of evidence, we investigated differences in the geographic distribution of return period thresholds, instead of the exact return periods of individual stations. In the present-day climate, regions estimated by the CMIP6 ensemble to experience noncompensable heat more frequently than 1-in-100 years contain 86.9% of weather stations with the same estimated return period. This excludes Europe and Japan due to known or identified problems with the model projections. Regions estimated to experience decadal return periods contain 70.1% of weather stations with return periods under the decadal threshold. Under increasing global average temperatures, the overlap between the two sets of results increases to 94.8 and 97.4% for the 1-in-100-year event and 87.8 and 96.9% for the 1-in-10-year event at 2° and 3.5°C respectively. We suggest that the independent agreement in the broad geographic distribution of high-frequency extremes across all six warming regimes is a strong argument for the fidelity of both results. This said, the two excluded regions are notable exceptions that must be explored.

The European continent was excluded from the above comparison because of the inability of the CMIP6 ensemble to capture the marked increase in heat extremes suggested by observational trends in that region. This is a known deficiency of global climate models already discussed at length above. The second exception is the lack of noncompensable heat exposure in Japan, which contains a large number of weather stations exhibiting return periods more frequent than 1-in-10 years given 2°C of global average temperature increase but return periods less frequent than 1-in-100 years in the CMIP6 projections across all warming levels. The CMIP6 models have a known low bias in specific humidity across the Japanese isles, driven by the difficulty with which GCMs simulate meridional moisture flux convergence in that region (*51*). This suggests that the bias could exhibit nonstationary characteristics and therefore may not be appropriately resolved via applied bias correction approaches; however, further investigation is needed to confirm the precise driver.

Last, there are 17 weather stations across Central Asia that exhibit more frequent than 1-in-100-year return periods not matched by the CMIP6 ensemble. There are several possible reasons for this. For example, poor data quality in the region (*47*), rapid urbanization, and recent decreases in some types of aerosol concentration (*52*) could be confounding the weather station results. There is also evidence supporting substantial temperature biases across Central Asia in both CMIP5 and CMIP6 (*46*, *53*) and across reanalysis products including European Centre for Medium-Range Weather Forecasts Reanalysis v.5 (ERA5) due to sparseness of available observational data (*54*). Given the *P* value of 0.05 used in the statistical analysis here, we expect roughly 5% of all stations to exhibit spurious trends, which, given the small number of stations in Central Asia, could also account for a proportion of the difference in results in this case.

## DISCUSSION

In this study, we investigate the global emergence of an empirically supported noncompensable heat limit. We demonstrate that the geographic range and frequency of noncompensable heat extremes will increase rapidly, given only moderate continued increase in global average temperatures. This implies that, in the near future, a substantial portion of the world’s population will be exposed to these noncompensable environmental conditions.

When considering the implications of these findings, we must take into account the multiple observations of noncompensable heat stress in the historical record, largely in high-population areas with low air-conditioning penetration (*4*) and the limited corresponding record of mass-casualty heat events among healthy human beings. Recent multicountry studies have demonstrated only a small increase, and in some regions a decline, in heat-related mortality (*55*–*58*) despite the well-established increase in frequency and severity of heat extremes over the past several decades discussed here and at length elsewhere (*24*). Behavioral and physiological heat adaptations are a critical confounding variable in interpreting the implications of projected future environmental conditions (*11*). In particular, there are many available forms of adaptation outside of air conditioning, including adjusting behavior (such as slowing, stopping, and/or seeking refuge in shade, water, or other naturally cooled environments) (*14*), electric fan use (*15*), aerobic training, and acclimatization (*13*). In addition, there are physiological factors that can decrease resiliency among specific populations, including advanced age (*59*), preexisting illnesses that result in compromised immune systems or reduced cardiovascular function (*60*), and the detrimental mental health impacts of extreme heat (*61*, *62*).

Combinations of epidemiological data and future temperature projections from climate models have been used elsewhere in an attempt to implicitly incorporate the impact of these behavioral and physiological adaptations into projections of future heat-driven mortality and morbidity. This includes the estimation of country-level temperature-related mortality damage functions (*63*), of trade-offs between future economic growth and excess heat-related mortality (*64*–*65*) and of subcountry-level heat-related mortality risk under different warming scenarios, for example, (*66*–*68*). However, these projections are predicated on the assumption that the historical relationship between environmental conditions and population vulnerability characteristics will remain constant or evolve smoothly, an assumption, which we know that a priori is not true as both behavioral and physiological adaptation will change over time and exhibit limits in the level of heat stress that they can mitigate (*13*). As a result of the changing relationship between heat and mortality, translating exposure to specific environmental conditions into precise mortality and morbidity impacts is very difficult.

This does not, however, mean that projections of future environmental conditions alone cannot be useful tools for understanding the risks of mitigation failure or for planning necessary adaptation efforts. In particular, even if the precise mortality and morbidity impacts are not quantifiable a priori, understanding the emergence of critical thermal limits can help policymakers inform their adaptation responses by identifying events that could substantively change the nature of impacts on the populations they are responsible for. Noncompensable heat is one such critical threshold.

Given that the noncompensable heat limit was calculated from experiments conducted on participants from a “warm-summer humid continental” (Dfb) climate zone (*12*), the lack of corresponding mortality or morbidity outcomes highlighted above is expected, as, to date, these extremes have only occurred in the hottest regions of the world, which are home to populations exhibiting substantial physiological and behavioral heat adaptation to heat extremes. However, climate change is driving the emergence of extreme events far outside of the range of historical experience across the world (*17*); in most locations, behavioral adaptation is tailored to historical experience (*17*), and physiological heat adaptation is a process that requires weeks of exposure to elevated temperatures (*13*). Hence, the increase in the geographic range of noncompensable heat events described above could cause a discontinuity in the historical relationship between heat and mortality for large parts of the world in the near term. There is a real risk of hundreds of millions of people being exposed to noncompensable heat as part of an extreme event before they are sufficiently physiologically and behaviorally heat-adapted to avoid attendant increases in mortality and morbidity.

We stress that the single threshold investigated here is most representative for mid-latitude populations and that further investigation will be necessary to better understand how the noncompensable heat limit changes as a function of subject acclimatization and as a result of the influence of other environmental factors including radiative load (e.g., sunlight), forced convection (e.g., wind speed), and duration of exposure (*12*). Nonetheless, our findings contribute to a more robust understanding of when and where differing critical thermal limits could be breached, so that policymakers can ensure the near-term sufficiency of heat action and adaptation plans based on the unique vulnerabilities of the regions for which they are responsible. Assuming that temperature-driven morbidity and mortality will continue to evolve following historical patterns, even in the near term, or that critical thermal limits will not be passed barring substantial increases in global average temperature (as with the 35°C wet-bulb threshold) will lead to myopic preparation for future heat risk, and so unnecessarily increase, perhaps markedly, the impacts of near-term climate change.

## MATERIALS AND METHODS

For the purposes of this analysis, we defined noncompensable heat stress as more than 6 hours of exposure to environmental conditions exceeding the physiological limits established in laboratory experiments conducted as part of the Pennsylvania State University Human Environmental Age Thresholds (PSU HEAT) project described in Vecellio *et al.* (*12*). The data provided by Vecellio *et al.* were converted to a heat threshold by fitting a quadratic function to the average noncompensable conditions observed across all participants for each of the six individual experiments conducted as part of their study (Fig. 1). The rationale behind the choice of a 6-hour exposure time was as follows: A healthy human core temperature ranges from 36° to 37°C. Death occurs given core temperatures greater than 43°C (*1*). As measured in-laboratory, when exposed to noncompensable heat, core temperature rises at an average of about 1°C/hour (*16*). Given at least 6 hours of exposure to noncompensable heat, therefore, a healthy, nonheat-adapted human being could see their core temperature rise to lethal levels. To investigate the sensitivity of our results to a changing duration of exposure, we also calculated return periods for 9- and 12-hour periods of exposure (see the Supplementary Materials).

**Fig. 6.**
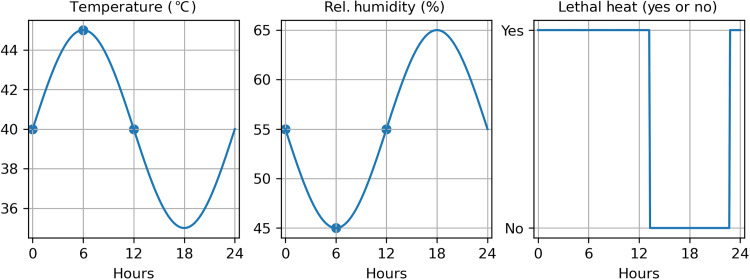
Method for extrapolating hourly temperature from daily modeled statistics. Temperature and relative humidity are approximately sinusoidal on the daily scale, allowing the estimation of high-frequency time series for each model point by fitting a sinusoid to daily maxima, minima, and means.

To examine the emergence of this noncompensable heat stress exposure in the future, we investigated statistical trends in weather station observations and climate model projections of annual temperature block maxima (the 6-hour period with the largest average noncompensable heat value for a given location and year). Weather station data were taken from the HadISD v3.3.0.2022f database, which is a quality-assured, global subdaily dataset based on the ISD dataset from National Oceanic and Atmospheric Administration’s National Centers for Environmental Information (NCEI) (*18*). Climate model data were taken from 17 members of the CMIP6 ensemble of ESMs/AOGCMs (see Table 1 below). The frequency with which meteorological observations are recorded varies from station to station, and, in some cases, hourly data are not available. In addition, hourly CMIP6 data are not always available and are computationally expensive to work with. To ensure a uniform time frequency across all data sources, we used an extrapolatory methodology to estimate hourly data from daily statistics—the mean, maxima, and minima of temperature and relative humidity. By assuming that temperature and relative humidity are sinusoidal on the daily scale, we can estimate high-frequency time series for each model point or observational station by fitting a sinusoid to daily maxima, minima, and means. The fitting procedure is straightforward as we only require the amplitude, assuming that the frequency is 24 hours and the relative phase between the two variables is 180° (Fig. 6). This method has been validated elsewhere (*69*).

**Table 1.** CMIP6 models used in analysis.

Model name	Horizontal resolution
ACCESS-CM2	1.9 × 1.3
ACCESS-ESM1-5	1.9 × 1.3
CanESM5	2.8 × 2.8
CNRM-CM6-1	1.4 × 1.4
CNRM-CM6-1-HR	0.5 × 0.5
CNRM-ESM2-1	1.4 × 1.4
EC-Earth3-Veg-LR	1.1 × 1.1
FGOALS-g3	2.0 × 2.0
GFDL-CM4	1.3 × 1.0
INM-CM4-8	2.0 × 1.5
INM-CM5-0	2.0 × 1.5
IPSL-CM6A-LR	2.5 × 1.3
MIROC6	1.4 × 1.4
MIROC-ES2L	2.8 × 2.8
MPI-ESM1-2-HR	0.9 × 0.9
MPI-ESM1-2-LR	1.9 × 1.9
MRI-ESM2-0	1.1 × 1.1

We then used hourly temperature and the respective relative humidity values across both datasets to calculate a scalar distance from the noncompensable heat function discussed above, such that a distance of 0 indicates a point on the function, negative values indicate compensable heat, and positive values indicate noncompensable heat. Last, scalar distances are normalized to a binary scale indicating noncompensable (1) or compensable (0) temperatures.

### Weather station analysis

During the production of the HadISD dataset, strict station selection criteria are applied, and a suite of quality control tests are conducted on major climatological variables (*47*). Nonetheless, additional quality control measures were required to prepare the data for analysis. We first removed all nonland-based stations, all stations north of 60°N and south of 60°S, all stations missing more than 50% of their data between 1970 and 2020, and, last, any stations where the 6-hour period with the highest average noncompensable heat value between 1950 and 1990 was larger than any period after 2000 (*10*). This reduced the available number of stations from 9555 to 4209. To account for erroneous data arising from instrumental or observer error that could otherwise bias the extraction of long-term trends from station data, we also considered data homogeneity. The pairwise homogeneity of HadISD data has been assessed elsewhere using the Menne-Williams algorithm (*70*). Stations for which the pairwise homogenization algorithm could not be run and, therefore, for which inhomogeneity values were not available or for which temperature inhomogeneity values are greater than 1°C were removed from the analysis, reducing available stations from 4209 to 3724.

Of the remaining 3724 quality-assured station datasets, 2110 were missing less than five consecutive observations, and 1614 were missing more. For those missing less than five consecutive observations, missing data were filled using nonlinear regression of the remaining station time series. For those missing five or more consecutive observations, the missing data were filled using a simplified adaptation of the algorithm in Tarvido and Berti (*71*), wherein, for each station, the three geographically closest stations with complete datasets were identified, and the missing data were interpolated using multivariate linear regression with the identified station time series as predictors. Regression-based methods have been demonstrated to produce more robust results compared to within-station or between-station methods when backfilling temperature observations (*71*). If there were not three stations with complete time series within a 100-km radius of the station in question, then that station was removed from the dataset. This process produced a dataset of 2512 stations with complete annual block maxima observations between 1970 and 2020, which was used as the basis for our analysis. Annual block maxima were examined for autocorrelation before analysis.

To extrapolate observed trends in the scalar value of annual noncompensable heat maxima, we fit a stationary GEV distribution to annual 6-hour block maxima extracted from each remaining weather station using the Nelder-Mead algorithm for maximum likelihood estimation and performed a Kolmogorov-Smirnov (KS) test to ensure that the block maxima could be appropriately modeled by such a distribution at a *P* = 0.05 significance level. Both the GEV fit and the KS test were performed using a Python package specifically developed for the analysis of climate extremes (*72*). The cumulative probability density function for the GEV distribution can be given as follows:

$$F(x;\mu ,\sigma ,\xi )=\exp\left\{-{\left[1+\xi \left(\frac{x-\mu }{\sigma }\right)\right]}^{-1/\xi }\right\}$$ (1)

Only two stations failed the KS test and, hence, were removed. Having identified the stations that can be modeled by GEV distributions, we then fit both stationary and nonstationary GEV distributions to each. For the nonstationary distributions, the location variable was parameterized as a linear function of global average temperature increase

$$\xi (T_{t})=a_{1}+a_{2}(T_{t})$$ (2)

A log-likelihood ratio test was then performed for each station to test the goodness of fit of both distributions. Under this test, the difference of the log likelihoods of the data under the two models is calculated to obtain the Log-Likelihood Ratio (LLR) statistic, Λ, which is then compared to a chi-squared distribution with degrees of freedom equal to the difference in the number of parameters between the two models. Weather stations where the nonstationary GEV fit results in a statistically significant improvement in goodness of fit at the *P* = 0.05 level were retained

$$qP(\text{Λ}=c|H_{0})+P(\text{Λ}<c|H_{0})=\alpha$$ (3)

Having performed both KS tests and log-likelihood ratio tests, the number of stations that can be acceptably modeled using a nonstationary GEV distribution was reduced from 2512 to 1410. With the remaining 1410 stations, the nonstationary GEV distributions were used to calculate the return period for an event with a scalar noncompensable heat magnitude greater than 0, indicating a noncompensable event, under global average temperature increases of 0.5°C increments between 1° and 3.5°C.

### Climate model analysis

There are some concerns related to the use of coarse-grain climate models for conducting local heat extreme projections, particularly when looking at changes in extremes relative to a reference period against which you also bias-correct (*73*). To address these concerns, we bias-corrected and statistically downscaled the raw CMIP6 data following the Inter-Sectoral Impact Model Intercomparison Project (ISIMIP) framework and using the W5E5 reanalysis product, a merged dataset combining WATCH Forcing Data Methodology Applied to ERA5 Reanalysis (WFDE5) data over land with ERA5 data over the ocean (*74*–*78*). The ISIMIP framework is specifically designed to downscale ESM ensembles to derive projections of the impacts of climate change across multiple temporal and spatial scales and has been applied widely and successfully to questions of future temperatures and health (*79*–*81*). All models were downscaled to a resolution of 0.5° by 0.5°.

Note that systemic issues with cloud representation cause some of the CMIP6 models to have unrealistically high climate sensitivities (*82*). We therefore structured our projections around common levels of observed surface warming, instead of common time periods along a forcing scenario—specifically, for each model, data were extracted for the three-decade period where mean global average temperatures first reach the indicated warming level. Having followed the procedures above, return periods for noncompensable heat stress were calculated directly from ensemble observations, treating each ensemble member as an independent sample of 30 years of observations from a given climate state. The list of models can be found in Table 1.
