# Supplementary material for: Observational and model evidence together support wide-spread exposure to noncompensable heat under continued global warming
Source: Sci Adv. 2023 Sep 8;9(36):eadg9297. doi: 10.1126/sciadv.adg9297 (PMC10491292; doi:10.1126/sciadv.adg9297)
Supplement: Supplementary file 1 — Figs. S1 to S3 [file sciadv.adg9297_sm.pdf]

Science Advances
AAAS

Supplementary Materials for

Observational and model evidence together support wide-spread exposure to noncompensable heat under continued global warming

Carter M. Powis *et al.*

Corresponding author: Carter M. Powis, carter.powis@ouce.ox.ac.uk

Sci. Adv. **9**, eadg9297 (2023)
DOI: 10.1126/sciadv.adg9297

This PDF file includes:

Figs. S1 to S3

Supplementary Materials for: Observational and model evidence together support wide-spread exposure to noncompensable heat under continued global warming

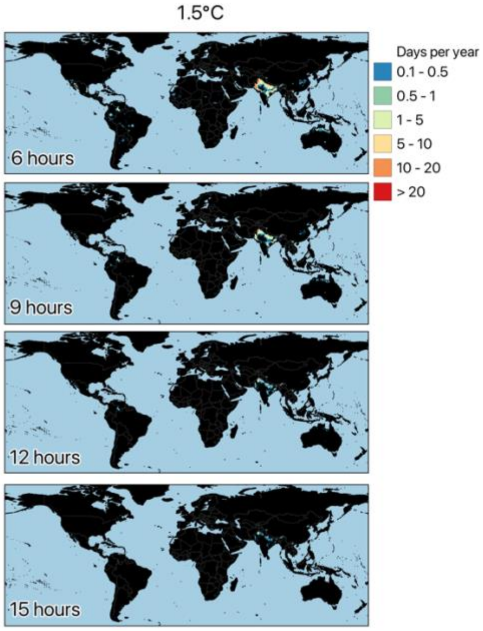

Supplementary Figure 1 (S1): Projections of non-compensable heat events under 1.5C global warming. Projections derived from a 16-member downscaled and bias-corrected CMIP6 ensemble. Panels show return periods assuming 6, 9, 12, and 15 hours of exposure.

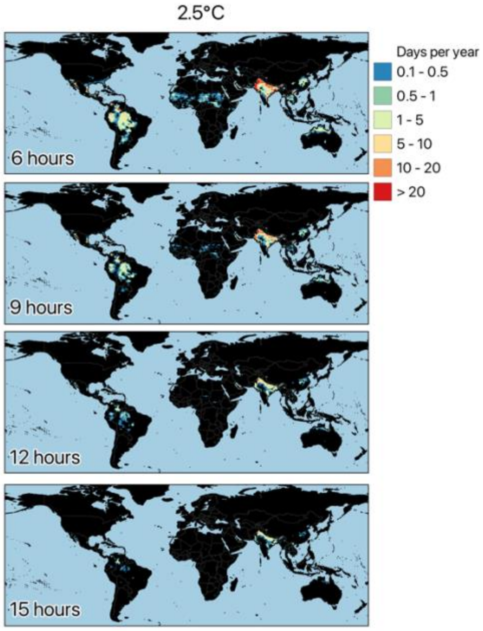

Supplementary Figure 2 (S2): Projections of non-compensable heat events under 2.5C global warming. Projections derived from a 16-member downscaled and bias-corrected CMIP6 ensemble. Panels show return periods assuming 6, 9, 12, and 15 hours of exposure.

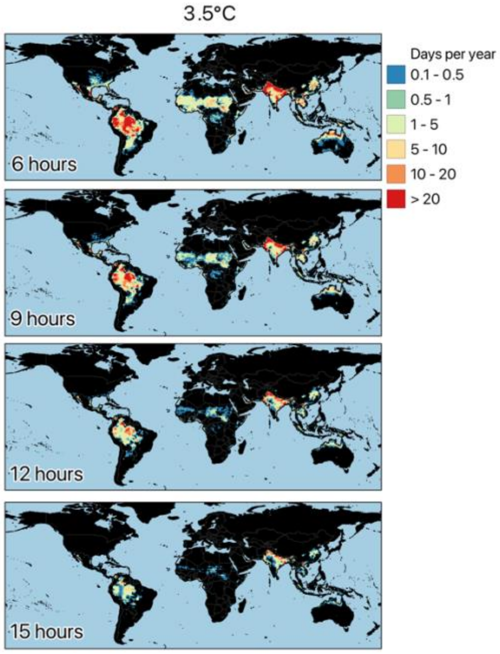

Supplementary Figure 3 (S3): Projections of non-compensable heat events under 3.5C global warming. Projections derived from a 16-member downscaled and bias-corrected CMIP6 ensemble. Panels show return periods assuming 6, 9, 12, and 15 hours of exposure.
